# Has the Rate of CD4 Cell Count Decline before Initiation of Antiretroviral Therapy Changed over the Course of the Dutch HIV Epidemic among MSM?

**DOI:** 10.1371/journal.pone.0064437

**Published:** 2013-05-27

**Authors:** Luuk Gras, Ronald B. Geskus, Suzanne Jurriaans, Margreet Bakker, Ard van Sighem, Daniela Bezemer, Christophe Fraser, Jan M. Prins, Ben Berkhout, Frank de Wolf for the ATHENA national observational cohort

**Affiliations:** 1 Stichting HIV Monitoring, Amsterdam, The Netherlands; 2 Department of Clinical Epidemiology, Biostatistics and Bioinformatics, Academic Medical Centre of the University of Amsterdam, Amsterdam, The Netherlands; 3 Department of Medical Microbiology, Centre for Infection and Immunity Amsterdam (CINIMA), Academic Medical Centre of the University of Amsterdam, Amsterdam, The Netherlands; 4 Department of Infectious Disease Epidemiology, Imperial College School of Medicine, London, United Kingdom; 5 Department of Internal Medicine, Division of Infectious Diseases, Tropical Medicine and AIDS, and Centre for Infection and Immunity Amsterdam (CINIMA), Academic Medical Centre of the University of Amsterdam, Amsterdam, The Netherlands; 6 Cluster of Infectious Diseases, Department of Research, Public Health Service of Amsterdam, Amsterdam, The Netherlands; University of Texas Health Science Center San Antonio Texas, United States of America

## Abstract

**Introduction:**

Studies suggest that the HIV-1 epidemic in the Netherlands may have become more virulent, leading to faster disease progression if untreated. Analysis of CD4 cell count decline before antiretroviral therapy (ART) initiation, a surrogate marker for disease progression, may be hampered by informative censoring as ART initiation is more likely with a steeper CD4 cell count decline.

**Methods:**

Development of CD4 cell count from 9 to 48 months after seroconversion was analyzed using a mixed-effects model and 2 models that jointly modeled CD4 cell counts and time to censoring event (start ART, <100 CD4 cells/mm^3^, or AIDS) among therapy-naïve MSM HIV-1 seroconverters in the Netherlands. These models make different assumptions about the censoring process.

**Results:**

All 3 models estimated lower median CD4 cell counts 9 months after seroconversion in later calendar years (623, 582, and 541 cells/mm^3^ for 1984–1995 [n = 111], 1996–2002 [n = 139], and 2003–2007 seroconverters [n = 356], respectively, shared-parameter model). Only the 2 joint-models found a trend for a steeper decline of CD4 cell counts with seroconversion in later calendar years (overall p-values 0.002 and 0.06 for the pattern-mixture and the shared-parameter model, respectively). In the shared-parameter model the median decline from 9 to 48 months was 276 cellsmm^3^ for 1984–1995 seroconverters and 308 cells/mm^3^ for 2003–2007 seroconverters (difference in slope, p = 0.045).

**Conclusion:**

Mixed-effects models underestimate the CD4 cell decline prior to starting ART. Joint-models suggest that CD4 cell count declines more rapidly in patients infected between 2003 and 2007 compared to patients infected before 1996.

## Introduction

The higher the HIV-1 plasma level, the more likely progression to AIDS is [1] and the higher the chance of HIV-1 transmission [2]. Previously, we found an increasing trend over time in plasma HIV-1 RNA concentration at set-point, i.e., 9 to 27 months after HIV-1 seroconversion and an accompanying decreasing trend of CD4 cell count measured at viral set-point [3]. In the Amsterdam Cohort Studies (ACS), a higher replicative fitness of HIV-1 isolates obtained from participants with seroconversion in more recent years was found [4]. Some cohorts have reported a similar increase in HIV-1 RNA concentration and decrease in CD4 cell count at viral set-point over time [5]–[7], whereas others found no evidence for such changes [8], [9]. The effect of higher viral load and lower CD4 T-cell count at set-point on progression to AIDS or death is difficult to study in the combination antiretroviral therapy (cART) era as these endpoints are hardly observed in effectively treated patients. A surrogate marker of disease progression is the rate of CD4 cell decline in patients not on therapy. Since individuals with low CD4 cell counts are more likely to start antiretroviral therapy (ART), the presence of informative censoring needs to be considered. Mixed-effects models, the standard method of analysis of longitudinal data, can give biased estimates when censoring (here: the start of ART) is not at random and depends on unobserved CD4 cell counts [10]. Joint-modeling of longitudinal CD4 cell counts together with the censoring process can give unbiased estimates, under certain assumptions. Therefore, we investigated trends in CD4 cell count decline between 9 months and 4 years after HIV seroconversion, using regression models that make different assumptions about the censoring pattern.

## Methods

### Patient Selection

Data were obtained from men having sex with men (MSM) participating in the ACS or the AIDS therapy evaluation in the Netherlands (ATHENA) cohort. ACS recruitment started in 1984, mainly among MSM living in or around Amsterdam. Inclusion criteria varied over time [11]. The ATHENA cohort has been described elsewhere [12] and includes anonymized data obtained from treated and untreated HIV-infected patients, who have been followed in or after 1996 in any of the 25 HIV treatment centers and also includes ACS participants in care in or after 1996.

From both cohorts, MSM from Western-Europe and North-America were selected with a maximum interval between the last negative and first positive HIV-1 antibody test of 1 year. The day of seroconversion was estimated as midpoint between both tests. Also included were MSM with serological evidence of acute infection in which case the day of seroconversion was set 1 month prior to the date of the first positive test. All MSM with seroconversion before 1996 were from the ACS. At seroconversion, all patients were 16 years or older. Patients had at least one CD4 cell count between 9 and 27 months after seroconversion while being ART-naïve. Subtype infections other than subtype B were excluded. MSM without subtype determination were included, since in our cohort the prevalence of subtype B infection among MSM from Western-Europe or North-America is 95% and therefore highly likely [12].

### Ethics Statement

Informed written consent is obtained from all ACS participants, and the study has been approved by the Medical Ethical Committee of the Academic Medical Centre. Ethical approval in the ATHENA cohort is not obtained as data are collected from patients as part of HIV care. Patients can opt-out after being informed by their treating physician of the purpose of collection of clinical data.

### Outcome

CD4 cell counts in peripheral blood measured between 9 months and 4 years after seroconversion were used to model CD4 cell count decline. The 4-year threshold ensured a similar follow-up period for subjects seroconverting in early and in later years. Only CD4 cell counts obtained from samples taken prior to any of the following 3 dates were included for analysis: date of first starting ART, first date CD4 cell count had dropped below 100 cells/mm^3^ (other studies [5], [8] have censored CD4 cell counts after this date because of the possibility of an accelerated decline), and the date 1 year prior to AIDS diagnosis (CD4 cell count decline may accelerate around this date [13], [14]).

### Statistical Analysis

Trends in CD4 cell decline over time were analyzed using 3 models with different underlying assumptions. All models assumed a linear decline and included a random slope and intercept for each patient. Age at seroconversion (linearly modeled and centered at 35 years of age) and timing of seroconversion (either in 3 categories 1984–1995, 1996–2002, 2003–2007, or as a continuous variable using natural splines with knots at January 1993, 2000 and 2005) were included as fixed covariates. cART was introduced in the Netherlands as standard of care in 1996 and this motivated our first group 1984–1995 (pre-cART era). The second cut-off was chosen a-priori so that each time period was sufficiently wide and included a sufficient number of patients. We used 3 different statistical models: 1) Mixed-effects models. These models can provide unbiased estimates if censoring only depends on the observed CD4 cell counts, given covariates included in the model (the censoring process is missing at random (MAR)). If instead censoring depends on unobserved CD4 counts, such as the underlying subject-specific CD4 cell count trajectories, estimates will be biased. Joint-modeling of longitudinal CD4 cell counts and the censoring process can give unbiased estimates under certain assumptions [15]. We used two joint-modeling approaches: 2) pattern-mixture models; and 3) shared-parameter models [16].

#### Pattern mixture model

Pattern-mixture models quantify the longitudinal outcome conditionally on the timing of the censoring event. The censoring event was either ART initiation, CD4 cell count dropping below 100 cells/mm^3^ or an AIDS diagnosis (in which case CD4 cell counts were censored from the date 1 year before the AIDS diagnosis). We used seven distinct patterns: censoring between 9–21, 21–27, 27–33, 33–39, 39–48 months, lost to follow-up (including suicide). A seventh pattern was no censoring event within 48 months. The basic probability structure of the model is:
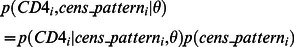
(1)With i an arbitrary individual and 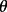 a categorical variable representing the 3 seroconversion periods 1984–1995, 1996–2002 and 2003–2007. The joint distribution in (1) is obtained by multiplying pattern-specific probabilities of CD4 trajectories 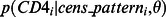, modelled via a random effects model, by weights according to the probability distribution, 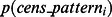. The parameter estimates of the CD4 trajectories, and their dependence on 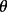, are obtained via likelihood maximisation. The weights from the second term. are obtained by dividing the number of patients in each pattern and in each period by the total number of patients in each period [17].

#### Shared parameter model

The basic probability structure of the model is:
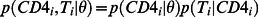
(2)with i and 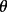 as above and T_i_ the time to the censoring event.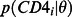 in (2) represents a longitudinal random effects model for the longitudinal CD4 cell counts and 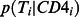 a survival model for the time to censoring due to ART initiation, CD4 cell count dropping below 100 cells/mm^3^ or an AIDS diagnosis. Individuals that had complete follow-up were treated as censored cases. The survival model was regressed on the underlying CD4 cell count and age. We used a Weibull accelerated failure time model, but a Cox proportional hazards model gave similar results. In the shared-parameter model, the association between the censoring times and CD4 cell counts is modeled via a set of random effects. Conditional on the random effects, the longitudinal CD4 cell counts are independent of the censoring time, CD4 cell counts were cube root transformed to comply with normality assumptions. Both terms contribute to the likelihood, hence a joint likelihood was maximized.

Averaged mean estimates from the pattern-mixture model were estimated using PROC MIXED in SAS 9.2 (SAS Institute, Cary, NC). The obtained standard errors are slightly underestimated because the size of the patterns are assumed fixed. To correct for the uncertainty of pattern sizes, corrected standard errors can be obtained using the delta method [17], [18]. Shared-parameter modeling was done using the R (R Development Core Team 2010) package JM [19]. SAS and R syntax is given in Supporting Information S1.

## Results

In total, 606 patients were included in the study: 111 with seroconversion between 1984 and 1995, 139 between 1996 and 2002, and 356 between 2003 and 2007. Of all MSM, 9 (8%) out of 120 with seroconversion between 1984 and 1995, 122 (47%) out of 261 with seroconversion between 1996 and 2002, and 165 (32%) out of 521 with seroconversion between 2003 and 2007, were excluded because either the date of ART initiation, the date CD4 cell count had dropped below 100 cells/mm^3^, or the date 1 year prior to diagnosis of AIDS was before 9 months after seroconversion. A last negative HIV test within 1 year of the first positive test was available for 524 patients. The remaining 82 patients had serological evidence of acute infection (3 seroconverters between 1984 and 1995, 15 between 1996 and 2002, and 64 between 2003 and 2007).

Baseline characteristics are shown in Table 1. Subtype was not determined in 330 patients (54%). The percentage of samples measured using a single-platform method gradually increased from 0% prior to 1997 to 94% in 2010. Timing of the first sample taken after 9 months after seroconversion was similar between the 3 seroconversion periods. Overall, the median first CD4 cell count at 9 months was 540 cells/mm^3^ but it decreased with later periods of seroconversion. The proportion of patients with a censoring event increased over time. ART was started in 295 patients, in 30 patients AIDS was diagnosed, and in 14 patients CD4 cell count declined to below 100 cells/mm^3^.

**Table 1:** Characteristics of 606 included MSM living in the Netherlands with seroconversion between 1984 and 2007.

	Year of seroconversion			
	1984–1995	1996–2002	2003–2007	Total
	N = 111	N = 139	N = 356	N = 606
	Median (IQR)	Median (IQR)	Median (IQR)	Median (IQR)
**Age at seroconversion (years)**	35.2 (29.7–42.1)	34.6 (30.2–41.1)	37.9 (31.6–43.8)	36.6 (30.6–43.1)
**First CD4 cell count 9–27 months after seroconversion (cells/mm^3^)**	580 (450–850)	550 (450–720)	510 (390–660)	540 (410–710)
**Months between seroconversion and first CD4 cell count**	10.3 (9.9–10.7)	10.7 (9.7–12.3)	10.5 (9.6–11.9)	10.4 (9.7–11.7)
**Number of included CD4 cell count measurements**	13 (10–14)	6 (4–8)	7 (4–9)	7 (4–10)
**Number of included CD4 cell count measurements**	13 (10–14)	6 (4–8)	7 (4–9)	7 (4–10)
	**N (%)**	**N (%)**	**N (%)**	**N (%)**
**Timing of censoring**				
9–21 months	6 (5%)	34 (24%)	76 (21%)	116 (19%)
21–27 months	4 (4%)	14 (10%)	44 (12%)	62 (10%)
27–33 months	12 (11%)	5 (4%)	29 (8%)	46 (8%)
33–39 months	4 (4%)	10 (7%)	33 (9%)	47 (8%)
39–48 months	10 (9%)	12 (9%)	46 (13%)	68 (11%)
No censoring within 48 months	67 (60%)	58 (42%)	116 (33%)	241 (40%)
Lost to follow-up 9–48 months	8 (7%)	6 (4%)	12 (3%)	26 (4%)
**Reason of censoring within 48 months**				
Start ART	14 (39%)	68 (91%)	213 (93%)	295 (49%)
AIDS diagnosis	15 (42%)	5 (7%)	10 (4%)	30 (5%)
<100 CD4 cells/mm^3^	7 (19%)	2 (2%)	5 (2%)	14 (2%)

IQR: Interquartile range, MSM: men who have sex with men.

**Figure 1:**
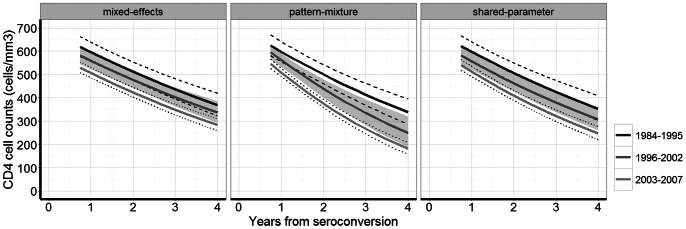
CD4 cell count for a typical patient (36 years of age), backtransformed to original scale by method of estimation. Dashed lines and shaded regions are 95% confidence intervals.

**Figure 2:**
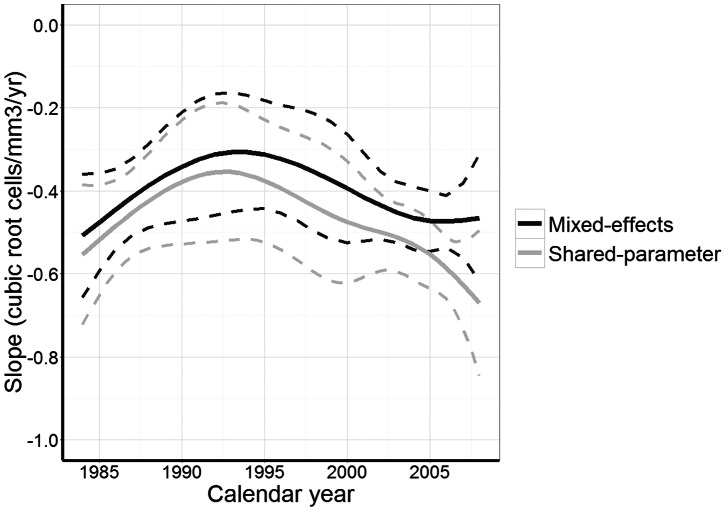
Mean slope (fat line) of CD4 cell count and 95% CI (dashed thin lines) in 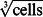/mm^3^/year according to calendar year of seroconversion estimated using a mixed-effects model and shared-parameter model.

Intercepts (estimated CD4 cell count at 9 months after seroconversion) were similar between the 3 models (see Table 2). All models showed a significant association of a lower CD4 cell count at 9 months with seroconversion in later calendar years. Estimates of the decline in CD4 cell count obtained with the mixed-effects model did not differ significantly between periods of seroconversion (overall p-value = 0.56). However, in the pattern-mixture model there was a significant association of an increasing CD4 cell count decline with seroconversion in later years (p = 0.002) whilst in the shared parameter model there was a borderline trend (p = 0.06). In both joint-models, the decline for 2003–2007 seroconverters was significantly higher than for 1984–1995 seroconverters. In the shared-parameter model, the difference was 0.12 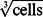/mm^3^/year (95% CI 0.00, 0.24). Estimates obtained with the pattern-mixture model showed a similar trend, although the rate of decline was steeper across all seroconversion periods compared to the shared-parameter model. In both joint models the decline did not differ significantly between seroconversion periods 1996–2002 and 2003–2007. Figure 1a–c shows the estimated decline in CD4 cell count graphically, on the original scale. When modeled using splines (Figure 2), the association between year of seroconversion and slope was borderline significant in the shared-parameter model (p = 0.06) but not in the mixed-effects model (p = 0.32). As expected, the estimates from the mixed-effects and the shared-parameter model differed least during the period when ART was not available. Shared-parameter model estimates suggest that the CD4 decline became smaller over time before 1993 and became larger thereafter. However, 95% confidence intervals were wide.

**Table 2:** Estimates of mean CD4 cell count at 9 months after seroconversion and rate of decline in CD4 cell count between 9 and 48 months using 3 methods.

	Mean CD4 cell countat 9 monthsafter seroconversion, 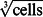 /mm^3^(95% CI)	Difference with2003–2007 (95% CI)	p-value (overall)	Mean slope, 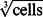 /mm^3^/yr(95% CI)	Difference in slope with 2003–2007(95% CI)	p-value (overall)
**Mixed-effects** **Model**			(0.0005)			(0.56)
1984–1995	8.52 (8.32, 8.72)	0.43 (0.20, 0.66)	0.0002	−0.42 (−0.50, −0.33)	0.05 (−0.05, 0.16)	0.33
1996–2002	8.35 (8.16, 8.53)	0.25 (0.04, 0.47)	0.02	−0.43 (−0.52, −0.33)	0.04 (−0.07, 0.15)	0.47
2003–2007	8.09 (7.97, 8.21)	0.00		−0.47 (−0.53, −0.41)	0.00	
**Pattern-mixture** **model**			(0.002)			(0.002)
1984–1995	8.55 (8.36, 8.73)	0.37 (0.19, 0.55)	0.0008	−0.49 (−0.60, −0.37)	0.28 (0.17, 0.40)	0.0004
1996–2002	8.42 (8.23, 8.61)	0.24 (0.05, 0.43)	0.03	−0.65 (−0.85, −0.45)	0.12 (−0.08, 0.32)	0.30
2003–2007	8.17 (8.06, 8.29)	0.00		−0.77 (−0.87, −0.67)	0.00	
**Shared-parameter model**			(0.002)			(0.06)
1984–1995	8.54 (8.34, 8.73)	0.39 (0.16, 0.62)	0.0008	−0.45 (−0.55, −0.35)	0.12 (0.00, 0.24)	0.045
1996–2002	8.35 (8.17, 8.53)	0.20 (−0.01, 0.42)	0.06	−0.49 (−0.60, −0.38)	0.08 (−0.04, 0.20)	0.19
2003–2007	8.15 (8.03, 8.26)	0.00		−0.57 (−0.64, −0.50)	0.00	

In all models age at seroconversion (as a continuous variable, centered at 36 years of age) was included as an interaction term both with the CD4 cell count at 9 months and the slope.

CI: confidence interval.

There was no significant association between age and the mean CD4 cell count 9 months after seroconversion in any of the 3 models. Nor did the mixed-effects and pattern-mixture models find an association between age and the slope of decline in CD4 cell count after 9 months (−0.03 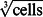/mm^3^/year, 95% CI −0.08, 0.02 and, 0.01 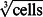/mm^3^/year, 95% CI −0.03, 0.05 per 10-year increase in age, respectively). The shared-parameter model estimated a stronger association between decline in CD4 cell count and older age; −0.06 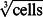/mm^3^/year per 10-year increase in age, 95% CI −0.12, −0.01.

For a typical seroconverter, i.e., an individual 36 years of age at seroconversion, with a median CD4 cell count at 9 months, the median decline between 9 and 48 months (see Table 3) in the pattern-mixture model changed from 286 CD4 cells/mm^3^ for 1984–1995 seroconverters (on average 88 cells/mm^3^/year) to 363 cells/mm^3^ for 2003–2007 seroconverters (112 cells/mm^3^/year). In the shared-parameter model the estimated median decline was smaller (83 cells/mm^3^/year for 1984–1995, and 90 cells/mm^3^/year for 2003–2007 seroconverters) whereas the mixed-effects model decline estimates were substantially smaller (between 75 and 77 cells/mm^3^/year across the 3 periods) compared to the pattern-mixture model estimates. Table 3 also shows that the pattern-mixture model estimated the median time from seroconversion to the 350 CD4 cells/mm^3^ threshold was 2.2 years for 2003–2007 seroconverters compared to 3.8 years for 1984–1995 seroconverters. This compares to 2.7 years and more than 4.0 years for the shared-parameter model.

**Table 3:** Estimated CD4 cell count decline (cells/mm^3^) between 9 and 48 months and time between seroconversion and decline to 350 CD4 cells/mm^3^ for a typical patient (aged 36 years) according to method of estimation and period of seroconversion.

		Method of estimation		
	Seroconversionperiod	Mixed-effects	Pattern-mixture	Shared-parameter
**CD4 cell count decline between 9 and 48 months (cells/mm^3^)**	1984–1995	−249	−286	−269
	1996–2002	−244	−346	−274
	2003–2007	−246	−363	−293
**Years between seroconversion and 350 CD4 cells/mm^3^**	1984–1995	≥4.0	3.8	≥4.0
	1996–2002	3.8	2.8	3.4
	2003–2007	3.0	2.2	2.7

## Discussion

Using joint-models, we found a trend of a steeper CD4 cell count decline before the initiation of antiretroviral therapy in HIV infected MSM since the early nineties. This suggests, together with the earlier reported higher viral fitness and the plasma viral load and lower CD4 cell count at viral set-point, an increasing trend in HIV virulence in MSM followed in HIV treatment centers in the Netherlands over time [3], [4]. Likewise, a recent meta-analysis concluded that virulence may have increased over the course of the HIV-1 pandemic [20]. The trend of a steeper CD4 cell count decline over time was only apparent when timing of censoring (because of ART initiation or disease progression) was jointly modeled with longitudinal CD4 cell counts and not when mixed-effects models were used. Mixed-effects models can provide unbiased estimates when the probability of the censoring event only depends on the observed CD4 cell counts (the MAR assumption), given covariates included in the model. Provided that the model for the censoring event is correctly specified, joint-models can provide unbiased estimates both when data are MAR, and when censoring is informative (when censoring depends on aspects of CD4 cell count that are not observed). The different results obtained from mixed-effects models and joint-models in this study, indicates that censoring CD4 cell counts due to ART initiation or disease progression is informative and estimates from standard mixed-effects models are probably incorrect. Estimation of parameters in joint models, compared to mixed-effect models, is more complex and joint models are currently not routinely used.

Using the shared-parameter model, we estimated an annual CD4 cell count decline of around 90 cells/mm^3^/year for 2003–2007 seroconverters. The pattern-mixture model estimated a steeper decline. The reported decrease among Italian patients included in the MASTER cohort [5] was less steep compared to our findings (approximately 55 cells/mm^3^/year with a trend towards steeper decline in more recent years for MSM). The Swiss HIV Cohort Study (SHCS), using the same methodology as the MASTER cohort, found no significant evidence for a changing rate of decline between 1986 and 2002 [8]. Analyses in both these studies were restricted to patients for whom at least 5 CD4 cell counts were available at least 200 days after the date of confirmed HIV infection. Furthermore, analyses were not restricted to patients for whom the date of seroconversion could reliably be estimated. Finally, estimates from the SHCS and MASTER cohort were obtained using mixed-effects models, without correction for informative dropout. The CASCADE collaboration [21] estimated the median time between seroconversion and reaching 350 CD4 cells/mm^3^ in 30–35-year old MSM with seroconversion after 1996 to be 3.94 years, similar to our mixed-effect estimate of 3.8 years for 1996–2002 seroconverters. Our joint-model estimates of 3.1 and 3.0 years were substantially lower indicating the MAR assumption may not hold. The CASCADE collaboration did not find evidence for an increased rate of CD4 cell count decline in more recent calendar years. CD4 cell count decline was jointly modeled with survival time. However, no correction for informative censoring due to ART initiation was made.

The reason for the less steep CD4 slope estimates and stronger effect of older age with CD4 cell count decline in the shared-parameter model compared to the pattern-mixture model is unclear, but an effect of age on either CD4 cell counts at 9 months on the slope, or both is to be expected, as shown by others [21], [22].

Estimates obtained using shared-parameter models allowing the estimated underlying CD4 cell count to have a different association with censoring due to the start of ART and censoring due to disease progression (censoring because of <100 CD4 cells/mm^3^, or 1 year prior to AIDS) were similar to those presented (results not shown). Furthermore, estimates from models censoring only CD4 cell counts because of ART initiation were also similar to our main results (results not shown).

Combined together, the ACS and ATHENA cohorts span almost the entire period of the Dutch HIV epidemic. A potential weakness is that ACS participants (all 1984–1995 seroconverters were ACS participants) might have different socioeconomic status, race, overall health status, or might otherwise be different to ATHENA participants. We attempted to minimize bias because of population changes by restricting inclusion to a very homogeneous population: MSM from Western-Europe or North-America and likely to be infected with subtype B. Most patients from both cohorts were seroconverters, and thus, had been tested for HIV before, suggesting a similar health seeking behaviour. Still, the possibility that our findings are the result of changes in patient characteristics over time cannot be excluded. The proportion of patients in our study identified through serological evidence of recent HIV-1 seroconversion increased from 3% between 1984 and 1995 to 18% between 2003 and 2007. This could have biased our results if patients went to seek medical care because of severe acute seroconversion illness and would otherwise not have been diagnosed. The number of symptoms during primary infection is associated with viral load set-point [23] and some specific signs with faster disease progression [24]. However, when these patients were excluded, similar results were obtained (results not shown).

Single-platform techniques to determine CD4 cell counts, gradually introduced in the late nineties, reportedly measure lower CD4 cell counts than dual-platform techniques [25]. We did not find a significant association between the standard CD4 cell count measurement technology at the time of the CD4 cell count measurement and the level of CD4 cell counts, and estimates in all 3 types of models remained similar (results not shown). However, this adjustment may have been incomplete as CD4 cell counts may have been measured with other techniques than the standard method. Factors influencing the number of measured CD4 cells which we could not control for include gating strategy, tobacco use and the time of day that the sample was taken [25]–[27].

Our study did not include patients who had started ART, or had a CD4 cell count below 100 cells/mm^3^ within 9 months or AIDS within 21 months after seroconversion. As we had to exclude a high proportion of seroconverters in the cART era for this reason, this may have biased estimates for the 1996–2002 and 2003–2007 periods. Patients starting therapy soon after infection, outside the setting of a randomized clinical trial, are more likely to have low CD4 cell counts and/or a more pronounced decline. Hence our estimates of the slope in 1996–2002 and 2003–2007 may have been upwardly biased. However, one could also argue that 1996–2002 and 2003–2007 estimates may be downwardly biased because patients with an early rapid CD4 cell count decline and starting ART before 9 months may have had a slower decline thereafter (because of regression to the mean effects) which is now not observed because of the early start of ART. Regression to the mean effects may have a dampening effect on bias resulting from exclusion of patients starting early treatment.

The probability of transmission of HIV is higher when the viral load is higher [28]. The duration of asymptomatic HIV infection increases with lower set-point viral load [14]. Together these factors determine the expected number of new infections an infected person will cause during the course of infection [29]. Evidence suggests that the level of set-point viral load may be largely determined by virus genotype [30] and that viral load between transmitter and recipient is correlated [31]–[35]. This may result in a selection of viral strains with higher set-point viral load because the infectious period is shortened in the cART era (transmission during successful ART is absent or near zero [36]). The proportion of transmitters with high set-point viral load among those transmitting HIV will increase and may lead, if viral load between transmitter and recipient is indeed correlated, to the observed increase in viral load and lower CD4 cell count at viral set-point [3] and a steeper decline in CD4 cell count.

Another explanation of our finding may be changes in host genetic factors or adaption of HIV-1 to host genetic factors [37] [38] or a rise in superinfection incidence, associated with a higher fitness [39]. Although dual infections can easily be missed, the incidence of 0% between 1984 and 1997 [40] and 1 to 1.5% between 1996 and 2007 in Amsterdam patients [41], [42] suggests that an increase over the years, caused by an increase in risk behavior [43], is unlikely to completely explain our findings.

In summary, to minimize bias it is important to jointly model timing of censoring because of ART initiation and/or disease progression in the analysis of longitudinal CD4 cell count trends. By doing so, we found a trend for a faster CD4 cell count decline in MSM with newly acquired HIV-1 infection between 2003 and 2007 compared to the pre-cART era. This could indicate an increase in HIV virulence over time. The higher rate of CD4 cell count decline, together with a lower CD4 cell count 9 months after seroconversion, results in a shorter time period between HIV infection and reaching the threshold of 350 CD4 cells/mm^3^.

## Supporting Information

Supporting Information S1 (DOC)
