# Supplementary material for: Has the Rate of CD4 Cell Count Decline before Initiation of Antiretroviral Therapy Changed over the Course of the Dutch HIV Epidemic among MSM?
Source: PLoS One. 2013 May 27;8(5):e64437. doi: 10.1371/journal.pone.0064437 (PMC3664616; doi:10.1371/journal.pone.0064437)
Supplement: Supporting Information S1 — (DOC) [file pone.0064437.s001.doc]

Data from 3 patients, categorical variable ‘DXXX’ represents timing of censoring as shown in Table 1 (7 categories), continuous variable ‘TDROPOUT’ represents timing of dropout in years from 9 months after seroconversion (‘TDROPOUT’=3.25 is 4 years after seroconversion) and ‘DROPOUT’ is a censoring indicator.

**ID YSERO TT CD4 CUBRTCD4 AGE10 DXXX TDROPOUT DROPOUT**

1 1984-1995 0.08277 850 9.4727 0.48549 6 3.25 0

1 1984-1995 0.33209 650 8.6624 0.48549 6 3.25 0

1 1984-1995 0.57044 990 9.9666 0.48549 6 3.25 0

1 1984-1995 0.80332 680 8.7937 0.48549 6 3.25 0

1 1984-1995 1.06907 650 8.6624 0.48549 6 3.25 0

1 1984-1995 1.31839 760 9.1258 0.48549 6 3.25 0

1 1984-1995 1.49647 640 8.6177 0.48549 6 3.25 0

1 1984-1995 1.74853 540 8.1433 0.48549 6 3.25 0

1 1984-1995 2.01154 580 8.3396 0.48549 6 3.25 0

1 1984-1995 2.21976 500 7.9370 0.48549 6 3.25 0

1 1984-1995 2.47131 540 8.1433 0.48549 6 3.25 0

1 1984-1995 2.71995 490 7.8837 0.48549 6 3.25 0

1 1984-1995 2.97404 430 7.5478 0.48549 6 3.25 0

1 1984-1995 3.22541 360 7.1138 0.48549 6 3.25 0

2 1996-2002 0.03543 300 6.6943 -0.05644 3 1.78 1

2 1996-2002 0.37696 280 6.5421 -0.05644 3 1.78 1

2 1996-2002 0.79795 180 5.6462 -0.05644 3 1.78 1

2 1996-2002 1.04726 240 6.2145 -0.05644 3 1.78 1

2 1996-2002 1.29932 300 6.6943 -0.05644 3 1.78 1

2 1996-2002 1.56233 260 6.3825 -0.05644 3 1.78 1

2 1996-2002 1.73767 260 6.3825 -0.05644 3 1.78 1

3 2003-2007 0.05000 440 7.6059 0.05123 4 2.00 1

3 2003-2007 0.29932 460 7.7194 0.05123 4 2.00 1

3 2003-2007 0.54863 330 6.9104 0.05123 4 2.00 1

3 2003-2007 0.89304 410 7.4290 0.05123 4 2.00 1

3 2003-2007 1.16080 340 6.9795 0.05123 4 2.00 1

3 2003-2007 1.31381 350 7.0473 0.05123 4 2.00 1

3 2003-2007 1.44769 380 7.2432 0.05123 4 2.00 1

3 2003-2007 1.71849 350 7.0473 0.05123 4 2.00 1

3 2003-2007 1.94589 260 6.3825 0.05123 4 2.00 1

**SAS syntax for the pattern-mixed model**

This code gives averaged intercepts and slopes for each seroconversion period, as shown in Table 2. The estimated are weighted according to the proportional size of each pattern in each seroconversion period (the percentages in Table 1 divided by 100). The obtained standard errors are slightly underestimated because the size of the patterns are assumed fixed. To correct for the uncertainty of pattern sizes, corrected standard errors can be obtained using the delta method [17,18].

**proc** **mixed** data=data1 noclprint method=ml cl;

class id ysero dxxx;

model cubrtcd4=dxxx*ysero tt*dxxx*ysero age10 tt*age10 / noint solution cl;

random intercept tt / subject=id type=un ;

estimate 'intercept 1984-1995' dxxx*ysero **0.054** **0.036** **0.108** **0.036** **0.090** **0.604** **0.072** **0** **0** **0** **0** **0** **0** **0** **0** **0** **0** **0** **0** **0** **0** / cl;

estimate 'intercept 1996-2002' dxxx*ysero **0** **0** **0** **0** **0** **0** **0** **0.245** **0.100** **0.036** **0.072** **0.086** **0.417** **0.043** **0** **0** **0** **0** **0** **0** **0** / cl;

estimate 'intercept 2003-2007' dxxx*ysero **0** **0** **0** **0** **0** **0** **0** **0** **0** **0** **0** **0** **0** **0** **0.213** **0.124** **0.081** **0.093** **0.129** **0.326** **0.034** / cl;

estimate 'slope 1984-1995' tt*dxxx*ysero **0.054** **0.036** **0.108** **0.036** **0.090** **0.604** **0.072** **0** **0** **0** **0** **0** **0** **0** **0** **0** **0** **0** **0** **0** **0** / cl;

estimate 'slope 1996-2002' tt*dxxx*ysero **0** **0** **0** **0** **0** **0** **0** **0.245** **0.100** **0.036** **0.072** **0.086** **0.417** **0.043** **0** **0** **0** **0** **0** **0** **0** / cl;

estimate 'slope 2003-2007' tt*dxxx*ysero **0** **0** **0** **0** **0** **0** **0** **0** **0** **0** **0** **0** **0** **0** **0.213** **0.124** **0.081** **0.093** **0.129** **0.326** **0.034** / cl;

estimate 'intercept 1984-1995 vs 2003-2007'

dxxx*ysero **0.054** **0.036** **0.108** **0.036** **0.090** **0.604** **0.072** **0** **0** **0** **0** **0** **0** **0** -**0.213** -**0.124** -**0.081** -**0.093** -**0.129** -**0.326** -**0.034** / cl;

estimate 'intercept 1996-2002 2003-2007'

dxxx*ysero **0** **0** **0** **0** **0** **0** **0** **0.245** **0.100** **0.036** **0.072** **0.086** **0.417** **0.043** -**0.213** -**0.124** -**0.081** -**0.093** -**0.129** -**0.326** -**0.034** / cl;

estimate 'slope 1984-1995 vs 2003-2007'

tt*dxxx*ysero **0.054** **0.036** **0.108** **0.036** **0.090** **0.604** **0.072** **0** **0** **0** **0** **0** **0** **0** -**0.213** -**0.124** -**0.081** -**0.093** -**0.129** -**0.326** -**0.034** / cl;

estimate 'slope 1996-2002 vs 2003-2007'

tt*dxxx*ysero **0** **0** **0** **0** **0** **0** **0** **0.245** **0.100** **0.036** **0.072** **0.086** **0.417** **0.043** -**0.213** -**0.124** -**0.081** -**0.093** -**0.129** -**0.326** -**0.034** / cl;

**run**;

**R syntax for the shared parameter model**

First a Cox proportional hazard model and a random-effects model on dataset ‘data1’ is fitted. The longitudinal model uses the data structure of multiple records per patient, as shown above. The Cox proportional hazards models uses one record per patient (dataset data1.id). The estimates obtained from the longitudinal and survival model are used as initial values for the shared-parameter model.

library("JM")

# CREATE DATASET WITH ONE RECORD FOR EACH PATIENT, TO BE USED FOR THE SURVIVAL MODEL

data1.id <-data1[!duplicated(data1$ID),]

# fit the linear mixed effects and survival submodels separately;

fitLME <- lme(CUBRTCD4 ~ TT * (YSERO + AGE10), random = ~ TT | ID, data = data1)

fitSURV <- coxph(Surv(TDROPOUT, DROPOUT ) ~ YSERO + AGE10, data = data1.id, x = TRUE)

# Fit Joint Model

fit.jm.weibull <- jointModel(fitLME, fitSURV, timeVar = "TT", method = "weibull-AFT-aGH", parameterization = "value", GHk=16)

summary(fit.jm.weibull)
